# Cardiorenal Syndrome: Challenges in Everyday Clinical Practice and Key Points towards a Better Management

**DOI:** 10.3390/jcm12124121

**Published:** 2023-06-18

**Authors:** Theodora Georgopoulou, Ioannis Petrakis, Kleio Dermitzaki, Christos Pleros, Eleni Drosataki, Georgios Aletras, Emmanouil Foukarakis, Eirini Lioudaki, Emmanuel Androulakis, Kostas Stylianou

**Affiliations:** 1 Department of Cardiology, Venizeleio General Hospital, 71409 Heraklion, Greece; theodgeo7@gmail.com (T.G.); aletras.george@gmail.com (G.A.); mfouk@hotmail.com (E.F.); 2 Department of Nephrology, University General Hospital of Heraklion, 71500 Heraklion, Greece; petrakgia@gmail.com (I.P.); ekderm@gmail.com (K.D.); xpleros@gmail.com (C.P.); elenidro2@hotmail.com (E.D.); 3 Renal Unit, King’s College Hospital NHS Foundation Trust, London SE5 9RS, UK; eirini.lioudaki1@nhs.net; 4 Royal Brompton Hospital/Harefield NHS Foundation Trust, London SW3 6NP, UK; e.androulakis@rbht.nhs.uk

**Keywords:** cardiorenal syndrome, anemia, hypochloremia, biomarkers

## Abstract

Under the term cardiorenal syndrome (CRS) falls an increasing number of patients who present with combined heart and kidney dysfunction. Despite the increasing knowledge concerning CRS pathophysiology, diagnosis, and treatment, many of the aforementioned aspects remain obscure in everyday clinical practice. Some of the challenges that clinicians face when they treat CRS nowadays is the need for a patient-centered management with early diagnosis, early intervention, the distinction of true kidney injury from permissive renal function deterioration during decongestion therapy, and the development of therapeutic algorithms to guide therapy.

## 1. Introduction

Over the last decades, an increasing number of patients have suffered from combined heart and kidney dysfunction largely known as cardiorenal syndrome (CRS). The term “cardiorenal syndrome” first appeared in November 1913, when Thomas Lewis proposed the presence of a close relationship between the heart and the kidney [1]. Since then, much progress has been achieved in the fields of CRS pathogenesis, classification, and therapy. However, critical problems remain in clinical practice regarding accurate patient classification and early CRS identification before significant organ damage occurs. Furthermore, the present research has not resulted in a practical, tailored, and evidence-based therapeutic approach.

CRS often coexists with anemia, which in turn has a negative impact on the heart and renal axis. The cardiorenal anemia syndrome (CRAS) is associated with a higher mortality and morbidity rate. Treatment options for this category of patients are currently restricted to intravenous iron and erythropoiesis-stimulating agents (ESAs).

In this review, we pinpoint the fields of CRS that remain poorly understood with an emphasis on CRAS, a relatively new subtype of the syndrome requiring specific management.

### 1.1. Cardiorenal Syndrome: Current Definition and Classification of the Syndrome

Following the first mention of the syndrome in 1913 [1], the National Heart Lung and Blood Institute working group attempted a more thorough characterization in 2004. This early definition was successful in explaining the bidirectional interaction that exists between the heart and the kidney, as well as how renal or cardiac disease leads to subsequent harm of the other organ, yet, the syndrome’s intricacy was not fully understood.

In 2008, Ronco et al. [2] defined CRS as “disorders of the heart and kidneys whereby acute or chronic dysfunction in one organ may induce acute or chronic dysfunction of the other”. In the same year, the working group of Acute Disease Quality Initiative (ADQI) proposed the classification of CRS into five distinct subtypes according to the primary organ insulted. When the primary injured organ was the heart, it was called CRS types 1 and 2. When the major organ was the kidney it was called CRS types 3 and 4. Organ injury was classified as acute CRS (types 1 and 3), or chronic CRS (types 2 and 4). CRS type 5 denotes the simultaneous dysfunction of both organs as a result of a systemic insult (for example, cirrhosis or sepsis) [3].

Hatamizadeh et al., based on the underlying pathophysiology and the main clinical manifestations, proposed that CRS could be divided into seven subcategories: (1) hemodynamic, (2) uremic, (3) vascular, (4) neurohumoral, (5) anemia- and/or iron-metabolism-related, (6) mineral-metabolism-related, (7) malnutrition–inflammation–cachexia-related [4].

The ADQI classification has some limitations in terms of clinical use. Its main shortcoming is that clinicians cannot distinguish whether a patient has renocardiac or cardiorenal syndrome in the majority of cases. Most patients come with an episode of acute heart failure (AHF) or, more frequently, an acute decompensation on the basis of chronic heart failure (CHF) with associated acute kidney injury (AKI), making a correct classification of the patient into CRS types 1, 2, or 3 challenging. Similarly, in the case of a renocardiac syndrome (CRS type 3 or 4), it can be difficult, if not impossible, to determine if the AKI preceded the occurrence of the cardiac injury. Furthermore, patients are classified in overlapping subcategories during the course of their disease, complicating categorization and care even further.

A combined assessment tool that classifies patients as having either a cardiorenal or a renocardiac syndrome would be a more effective classification approach. This classification would be based on the patient’s history and a detailed clinical examination combined with cardiac and renal ultrasound. For example, echogenic small kidneys may indicate a renal predominance for the CRS. In contrast, normal sized kidneys with compromised heart function in a cardiac ultrasound may indicate the heart as the main culprit. The response to the therapeutic approach could be of further help in this classification. For example, a creatinine decline in response to diuretic therapy could be interpreted as cardiorenal syndrome type 1 or 2. On the contrary, a creatinine increase during decongestion therapy could be interpreted as a renocardiac CRS, due to the overestimation of the true GFR and the masked kidney failure due to volume overload.

A classification of CRS patients into different hemodynamic profiles based on their clinical phenotype has been proposed [5]. This classification method utilizes tissue perfusion adequacy (cardiac output (CO) and effective circulation fluid volume (ECFV)) and the extent of pulmonary congestion (central venous pressure (CVP) or pulmonary capillary wedge pressure (PCWP)). Accordingly, patients are classified into four subcategories “wet or dry” and “warm or cold” [6]. This classification, despite its predictive value in determining the need for urgent intervention and its usefulness in guiding decongestion therapy, receives limited use in everyday assessment as it requires an interventional and complex measurement of hemodynamic indices.

***Key point 1:*** It becomes evident that a new classification system of CRS is required to categorize patients early in the course of the disease (cardiorenal or renocardiac) in order to deliver the appropriate therapy. A kidney ultrasound, echocardiogram, and the response to decongestion treatment may be helpful.

### 1.2. Preventing CRS: Is It Possible to Identify the Patients at Risk?

Despite the significant value of an early diagnosis of CRS, it becomes more and more evident that CRS prevention and early identification of the patients at risk have a key role in CRS management because a late diagnosis may be associated with irreversible morbidity and organ damage.

Potential predisposing risk factors for CRS development are under investigation. However, the identification of contributing risk factors for either cardiorenal or renocardiac syndrome is difficult because of their convergent mutuality [7]. Male gender, advanced age, arterial hypertension, diabetes mellitus (DM), prior history of surgery and atrial fibrillation (AF) constitute independent risk factors for developing AKI [8]. The prevalence of AKI and its severity increases among patients hospitalized in the intensive care unit (ICU) with the overall mortality being as high as 80% in this population [9]. Baseline kidney dysfunction has been shown to predict kidney failure. In the prospective outcomes study in heart failure (POSH study), which followed 299 patients with an LVEF >40%, the baseline serum creatinine (SCr) was found to be an independent risk factor of worsening renal function (WRF) [10]. Moreover, a history of prior CHF and SCr at admission >1.5 mg/dL have been associated with WRF among CHF patients [11,12]. A post hoc analysis of the Evaluation Study of Congestive Heart Failure and Pulmonary Artery Catheterization Effectiveness (ESCAPE trial) demonstrated that prior history of DM and hypertension were associated with an increase in SCr of >0.3 mg/dL [13]. Moreover, patients developing WRF (defined as an increase ≥0.3 mg/dL in the serum creatinine level compared with the value on admission) in the context of HF tend to be older and suffer from atherosclerotic disease. A fluctuating GFR is associated with a higher risk of reduced cardiac index, a need for intravenous inotropes and vasodilator therapy, and a significantly higher mortality rate. Albuminuria, a well-known risk factor for the development of cardiovascular disease, also increases the risk for AKI [14,15]. Predisposition for WRF due to cachexia or obesity has not been proven yet by epidemiologic studies [16]. Treatment-related factors such as a high dose of diuretics in patients with ADHF demonstrated a significantly higher rate of WRF [17].

***Key point 2:*** A prognostic tool that incorporates the presence of risk factors (e.g., age, gender, DM, AF, hypertension, albuminuria), clinical examination findings, and patient history in conjunction with cardiac and renal biomarkers is crucial in order to properly identify high-risk patients for developing a more severe and progressive form of CRS.

### 1.3. New Insights into CRS Pathophysiology and the Emerging Role of Serum Chloride

CRS is characterized by a plethora of interacting pathophysiologic mechanisms. Each pathophysiologic mechanism impacts discretely on CRS’s natural course. Central venous pressure (CVP) and intra-abdominal pressure (IAP) are of major importance. Each cardiac decompensation episode supposedly leads to increased CVP and reduced blood return from renal veins ultimately causing congestion within the kidneys. Kidney congestion in turn leads to a reduced renal plasma flow, reduced glomerular filtration rate (GFR), enhanced fluid retention, and eventually, reduced urine output and renal dysfunction [18]. Both changes in IAP and CVP correlate with alterations in renal function. An increase in IAP above 12 mmHg is critical for WRF [19]. Similarly, an increase in CVP above 6 mmHg is associated with WRF and increased all-cause mortality in a broad spectrum of patients with cardiovascular disease [20]. For almost a century, venous congestion was thought to significantly impact organ perfusion, with increases in venous pressure being related with end-organ damage and AKI [21]. Unfortunately, clinical indicators of peripheral congestion have been shown to be insufficient in identifying fluid overload and thus in supporting clinical decisions. CVP has long been thought to be linked to venous congestion. However, research has cast doubt on its usefulness because CVP values fluctuate based on patient position, the presence of mechanical ventilation, and other factors that alter intrathoracic pressure [22]. For the time being, there is no clear consensus on precisely determining renal venous pressure, with the scientific focus shifting to the combinatory use of echocardiographic measures. The VEXUS scoring system incorporates three parameters: inferior vena cava dimensions and respiratory fluctuation, as well as hepatic, renal, and portal veins’ waveforms utilizing pulsed wave Doppler. This scoring system has been linked to the development of AKI in postoperative cardiac surgery patients. The incorporation of such scoring systems into everyday clinical practice may considerably aid decision-making in challenging circumstances such as CRS with the option to, for example, discontinue fluid administration, decide to increase diuretic treatment, and manage AKI [23]. Intrarenal venous flow Doppler with a measurement of the renal arterial resistive index as an indicator of renal congestion has been linked to the development of AKI in patients after cardiac surgery. We feel that combining all of the above noninvasive and low-cost approaches with a thorough clinical examination could be quite beneficial in the care of CRS patients [24].

A reduced renal plasma flow results from a reduced cardiac output. A decreased renal plasma flow leads to intrarenal renin release, which in turn causes the constriction of renal capillaries, sodium retention, and vascular congestion. The validity of this theory has been weakened after the findings of the ESCAPE trial [25]. No correlation was found between baseline renal function and cardiac index (CI), something rather reasonable considering the high prevalence of diverse kidney pathologies that can be irrelevant to heart failure. Furthermore, an improvement in CI did not always result in an improvement in renal function [26]. However, in patients presenting with acute cardiogenic shock, an association between CI and the incidence and severity of AKI does exist, suggesting that the contribution of the low flow theory is stronger and more obvious in the acute setting [27].

The role of the renin–angiotensin–aldosterone system (RAAS) has been well established in CRS pathophysiology, contributing simultaneously to the progress of HF and the deterioration of kidney function [28]. Angiotensin II (Ang-II) is the major effector peptide of this system. Its increased excretion is attributed to increased renin levels after overactivation of the sympathetic nervous system [29]. Ang-II causes renal efferent arteriolar vasoconstriction, enhanced sodium reabsorption from the proximal tubules, increased aldosterone-mediated sodium reabsorption, and an overexpression of endothelin-1 (ET-1) [30]. In the heart, Ang-II induces cardiac hypertrophy and a contraction of vascular smooth muscle cells. It also contributes to the development of oxidative stress via reactive oxygen species (ROS) formation.

Oxidative stress and chronic inflammation play a significant role in the development of CRS. The increased production of ROS surpasses the body’s antioxidative capacity and is attributed to inflammation, ischemic injury, and venous congestion [31]. Ischemia, venous congestion, the activation of the SNS and RAAS induce an inflammatory cascade of proinflammatory cytokines (TNF-a, interleukin-6, interleukin-1) and the production of oxidative stress markers such as myeloperoxidase and nitric oxide, which result in tissue dysfunction [32]. Recently developed pharmacotherapies such as novel antidiabetic drugs (sodium-glucose cotransporter-2 inhibitors, SGLT2i) and finerenone may be able to offset the important role of oxidative processes in cardiorenal disorders [33]. These agents have demonstrated a significant antioxidant activity in preclinical and clinical studies. Furthermore, histological investigations demonstrated that dapagliflozin therapy decreased mesangial expansion, macrophage infiltration, and tubulointerstitial fibrosis [34]. Heerspink et al. assessed the levels of biomarkers in plasma samples from patients with T2D enrolled in a randomized clinical trial and found that treatment with canagliflozin decreased levels of tumor necrosis factor receptor-1 (TNFR1), interleukin-6 (IL-6), matrix metalloproteinase-7 (MMP7), and fibronectin-1 (FN1) levels compared with the glimepiride treatment, suggesting that canagliflozin can attenuate the molecular pathways related to inflammation and fibrosis [35]. Furthermore, a systematic literature review of 30 studies showed that treatment with an SGLT2i resulted in decreases of IL-6, C-reactive protein (CRP), TNF-α, and monocyte chemoattractant protein-1 (MCP-1) [36].

Recently, sodium chloride has been suggested as a potential cardiorenal biomarker [37]. Chloride is an anion responsible for preserving serum osmolarity along with sodium as well as fluid and acid–base balance, “competing” with serum bicarbonate. A low serum chloride concentration leads to the activation of sodium potassium chloride cotransporter (NKCC) in the thick ascending limb of the loop of Henle (TAL) and the sodium chloride cotransporter (NCC) in the distal convoluted tubule [38]. Consequently, hypochloremia leads to diuretic resistance, a mechanism of major importance in the pathogenesis and management of CRS. Hanberg et al. showed that chloride depletion is a key mechanism for diuretic resistance and neurohormonal activation [39]. Maaten et al. highlighted the role of chloride in renal salt sensing, by showing that low serum chloride was strongly associated with impaired decongestion in AHF [40]. Another pathway linking chloride with HF, diuretic resistance, and CRS, is the capacity of chloride to suppress renin secretion and of hypochloremia to increase renin excretion via the mobilization of COX-2 and prostaglandins [41]. Moreover, hypochloremia promotes renal vasoconstriction and GFR reduction independently of renal enervation [42]. In clinical practice, hypochloremia has been shown to be an important adverse prognostic marker associated with a higher risk of mortality in CHF patients [43]. In a study conducted by Grodin et al., it was shown that serum chloride levels were independently and inversely associated with long-term mortality. Interestingly, the prognostic impact of hyponatremia was blunted if associated with normal chloride levels [44].

***Key point 3:*** The examination of serum chloride may pave the way for new findings in the field of diuretic resistance and CRS. However, it is unclear if chloride is only a marker of the severity and prognosis of CRS or a distinctive therapeutic target.

### 1.4. Acute Tubular Injury vs. Permissive WRF: The Overestimated Role of Creatinine

There has been a lot of debate about the clinical significance of WRF, defined as a short-term absolute increase in serum creatinine in patients with ADHF. A major difficulty in the management of CRS is to distinguish between true WRF, due to acute tubular injury, from a rise in creatinine concentration as a result of effective decongestion. Recently, it has been shown that WRF is rather a transient phenomenon attributed to intensive decongestive therapy, not accompanied by true renal injury. Among patients with ADHF and reduced ejection fraction only half of the cases of in-hospital WRF persisted after one month. Moreover, patients experiencing WRF with successful decongestion were not at increased mortality risk at 180 days, whereas, in the case of WRF with persistent congestion, there was a heightened risk for poor clinical outcomes [45]. A Japanese study also showed that the absence of AKI criteria on admission indicated a good prognosis even in the presence of WRF during hospital stay, indicating that the AKI criteria, and not a simple rise in serum creatinine (WRF), should be used for the evaluation of kidney injury [46].

It becomes increasingly clear that WRF should always be assessed in parallel with its clinical context. If adequate diuresis is present, a rise in creatinine is associated with better long-term outcomes as shown by several studies such as a post hoc analysis of the DOSE-AHF trial. Indeed, an aggressive diuresis during admission for AHF with or without WRF was associated with better clinical outcomes whereas a drop in serum creatinine was paradoxically associated with worse clinical outcomes [47]. In the PROTECT study, WRF defined as a creatinine increase of >0.3 mg/dL was associated with a longer hospital stay and worse 30- and 90-day outcomes especially in patients with residual congestion at the time of renal function assessment [48]. Similarly, Stolfo et al. showed that WRF did not affect the prognosis of ADHF and on the contrary, when associated with BNP reduction, identified patients with adequate decongestion at discharge and favorable outcome [49]. The detection of intrinsic renal damage remains a challenge and requires the combination of a clinical assessment in association with a rise of specific urinary and plasma biomarkers.

***Key point 4****:* Permissive WRF is not associated with unfavorable renal and overall outcomes when it is accompanied by decongestion and enhanced diuresis.

### 1.5. Diagnosis of Acute CRS: The Need for a Panel of Multiple Biomarkers

There has been a lot of discussion concerning the controversial role of serum creatinine and eGFR in the timely diagnosis of acute kidney injury. It is clear now that serum creatinine changes become clinically obvious once severe damage has already occurred, although these changes continue to be the final gold standard indicator for renal dysfunction. In view of the known weaknesses of creatinine, researchers have turned their attention to other potential cardiorenal biomarkers.

#### 1.5.1. Cardiac Biomarkers

The cardiac troponins, cardiac troponin T (cTnT), and cardiac troponin I (cTnI) are well-studied specific biomarkers of myocardial injury and infarction correlating with ventricular remodeling after HF and increasing with the progression of HF. Therefore, they have a role in risk stratification and prognosis in patients with HF. They also predict cardiovascular and all-cause mortality in patients with CKD [50].

The two preferred biomarkers for HF are B-type natriuretic peptide (BNP) and N-terminal probrain natriuretic peptide (NT-proBNP), released from cardiomyocytes in response to atrial stretching and evoke a natriuretic and cardioprotective role. They both correlate with HF NYHA classification, left ventricular ejection fraction (LVEF), and ventricular pressure, thus contributing to prognosis and risk stratification of patients with HF [51]. Moreover, they correlate with renal dysfunction and predict cardiovascular and all-cause mortality in CKD patients, with the NT-proBNP being more sensitive [52]. Bosselmann et al. assessed the prognostic significance of several CV biomarkers in patients with systolic dysfunction and renal dysfunction. Interestingly, it was shown that all five CV biomarkers (including cTnT, proatrial natriuretic peptide, copeptin, proadrenomedullin, and NT-proBNP) had a prognostic significance for mortality risk, that did not interact with renal dysfunction and could be interpreted independently of eGFR [53]. Copeptin (the C-terminal part of arginine vasopressin peptide) is a biomarker of cardiovascular diseases and a significant predictor of mortality in patients with myocardial infarction [54]. Adrenomedullin (ADM) is produced in the adrenal medulla, vascular endothelial cells, and in the heart in response to physical stretch and is associated with pressure and volume overload. Mid regional proadrenomedullin (MR-pro-ADM) is a more stable molecule than ADM, thus being easier to be measured. MR-pro ADM is a significant predictor of morbidity in HF and correlates with the development and progression of CKD [55].

#### 1.5.2. Renal Biomarkers

Until now, creatinine has remained the principal biomarker of renal function that guides therapeutic decisions and determines the presence of AKI. Despite its wide use, a rise in creatinine levels follows several hours to days (depending on renal reserve or AKI extend) after the initial insult failing to timely diagnose the renal injury. Therefore, the identification of new biomarkers for the early detection of AKI has become an increasing need in clinical practice. Among identified biomarkers associated with kidney function, cystatin-C has been well studied. Cystatin-C is an endogenous cysteine proteinase inhibitor that is freely filtered in the glomerulus, completely reabsorbed by renal tubular epithelial cells and is found in urine only during tubular injury. Plasma cystatin-C can increase earlier than creatinine in early stages of AKI [56] and can detect small reductions in GFR. Apart from its role as a marker of kidney function, cystatin-C is also an independent risk factor for all-cause and cardiovascular mortality among elderly persons with or without CKD. Cystatin-C is also related to HF progression, cardiovascular events and death, thus being a potential predictor of cardiovascular complications in patients with atherosclerosis and coronary heart disease [57,58].

NGAL is a useful early marker for AKI, being able to diagnose the development of AKI up to 48 h prior to a clinical diagnosis, also correlating with AKI severity [59]. The value of serum NGAL in AHF was assessed in the AKINESIS study which found that plasma NGAL was not superior to creatinine for predicting WRF and therefore its use to diagnose AKI in AHF could not be recommended [60]. On the other hand, urinary NGAL may predict the development of WRF in AHF. Overall, the diagnostic utility of NGAL varies between different patient populations and is affected by comorbidities, timing of measurement, and cutoff values [61].

Kidney injury molecule-1 (KIM-1) is a transmembrane glycoprotein markedly expressed by the proximal tubule in response to renal injury, being a reliable predictor of AKI. It is also a predictor of disease progression in various cardiovascular diseases such as myocardial infraction and postcardiac surgery [62].

#### 1.5.3. Other Biomarkers

C-type natriuretic peptide (CNP) together with atrial (ANP) and B-type (BNP) natriuretic peptides make up the family of natriuretic peptides, a family of hormones involved in the regulation of blood pressure, electrolyte, and volume homeostasis [63]. Both ANP and BNP have been extensively studied during the past years, whereas CNP, the ancestral precursor from which these two molecules evolved, and urinary CNP have recently attracted the attention of research as emerging biomarkers in HF and renal injury. CNP is mainly expressed in the kidney but also in cardiomyocytes, vascular endothelium, and bone [64]. Plasma levels of CNP are typically low and CNP is thought to act as an autocrine or paracrine factor. Urinary CNP is predominantly derived from local renal production and the urinary CNP excretion rate reflects renal structural integrity and function. CNP lacks significant diuretic and natriuretic effects under normal circumstances but demonstrates antiproliferative and antifibrotic properties and also exhibits a vasodilating role, thus contributing to the regulation of vascular tone [65]. Urinary CNP levels have been shown to increase in patients with AHF, suggesting an activation of the renal natriuretic peptide system in HF. An elevation of the urinary excretion of CNP is probably attributed to increased renal interstitial pressure, renal tubular injury, hypoxia, and renal fibrosis. CNP correlates with prognosis, in the setting of AHF, being able to detect renal dysfunction in HF better than urinary NGAL and KIM-1 [64]. Urinary CNP excretion may represent a marker of early renal structural remodeling and underlying renal injury both acutely and chronically [66]. CNP also has a dominant role in HF with its plasma levels been increased in this setting and correlating with a high-risk group of patients with cardiovascular comorbidities and left ventricular dysfunction.

#### 1.5.4. Novel Diagnostic Methods

**Figure 1:**
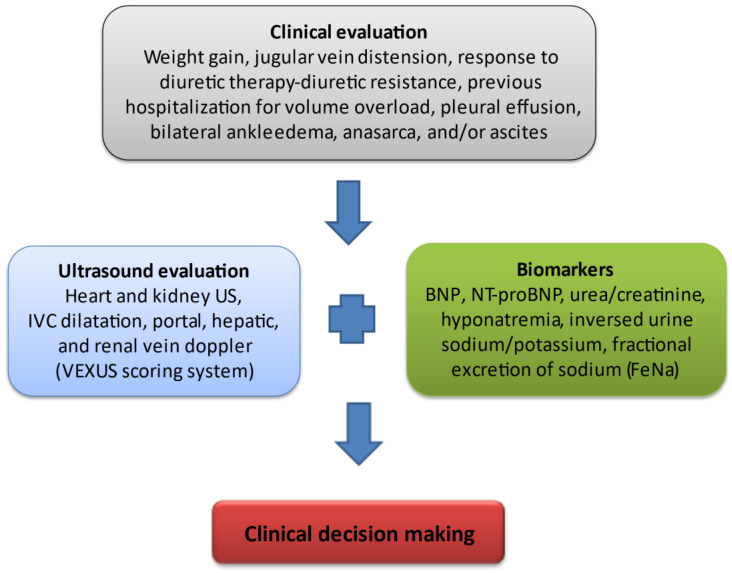
Assessment of venous congestion in CRS. Fluid overload has been clearly associated with adverse outcomes in critically ill patients such as end-organ damage and therefore, an increased incidence of acute kidney injury. Renal dysfunction developing in the setting of hypervolemia results from a decline in renal venous outflow and hence an increase in renal interstitial pressure. Novel methods of assessing venous congestion are much needed in order to establish effective and timely, appropriate decongestive treatment. Clinical evaluation is a key step for the assessment of volume overload including recurrent weight gain, the development of diuretic resistance (constantly increasing diuretic dose to achieve effective decongestion and/or the need for a combinatory use of diuretics with different modes of action for sequential nephron segment blockade), frequent hospitalizations in order to receive intravenous diuretic therapy, the presence of pleural effusion, peripheral oedema/anasarca, and/or ascites. Inferior vena cava (IVC) is the first venous compartment where congestion becomes apparent. Hepatic venous flow abnormality follows IVC distension with a subsequent development of portal vein pulsatility and renal venous flow Doppler abnormalities. All the above measurements constitute the VEXUS scoring system, with elevated levels if natriuretic peptides (B-type natriuretic peptide, N-terminal pro-brain natriuretic peptide) are signs of intravascular and intracardiac congestion. An increased ratio of blood urea nitrogen (BUN) to creatinine as well as dilutional hyponatremia are markers of the pathological activation of the renin–angiotensin–aldosterone system and sympathetic nervous system. The inversed urine ratio of sodium to potassium ratio as well as a constantly decreased fractional excretion of sodium in urine both depict a potential mechanism of diuretic resistance, therefore favoring the intensification of diuretic therapy.

Given that CRS is a syndrome with various clinical features, correct diagnosis can be extremely challenging in such patients. Artificial intelligence using expert-driven knowledge and specialized machine-based decision trees can help significantly towards this direction. Indeed, this was shown in a paper by Dong-Ju Choi et al., where an artificial intelligence clinical decision support system (AI-CCSS) presented a high diagnostic accuracy for heart failure [67]. Further development of such AI-based tools could be of significant importance in patients with CRS, where proper and early diagnosis is the key for optimal management (Table 1 and Figure 1).

***Key point 5:*** A panel of new biomarkers (in plasma and/or urine) and artificial intelligence support systems could aid in the early identification of CRS patients at risk of developing adverse outcomes.

### 1.6. Treatment and Management of CRS: Questions to Be Answered?

The therapeutic pillars in the management of CRS are decongestion, endogenous vasodilation, inotropic support, inhibition of the neurohormonal axis, and extracorporeal therapy. Intravenous loop diuretics are considered the gold standard of therapy although they have not been shown to improve survival or attenuate disease progression [68]. Despite the wide recommendation for systematic use of loop diuretics in the management of CRS so as to achieve aggressive diuresis, a gap of knowledge remains concerning their optimal use. First of all, data on diuresis in heart failure have been collectively extrapolated from patients with and without renal dysfunction. Therefore, the effect of loop diuretics on the cardiorenal axis has not been specifically studied.

The optimal dose of diuretics administered is dependent on the degree of renal dysfunction. Thus, various algorithms for dose adjustment of loop diuretics and thiazides (if used in conjunction) exist in order to guide decongestion [69]. However, dose adjustment for other diuretics in renal insufficiency has not been established. Another question concerns the mode of administration of loop diuretics, that is, continuous vs. bolus therapy. According to the DOSE trial (Diuretic Optimization Strategies Evaluation), a study that compared continuous vs. intermittent infusions of furosemide, there was no difference between these modalities in symptoms control or net fluid loss at 72 h. In addition, the group of patients receiving a higher dose of diuretics presented more frequently short-term WRF compared with the group receiving lower doses, but in the long term (60 days), no adverse outcomes were noticed, suggesting that a rise in creatinine in the context of decongestion should always be assessed in parallel with the clinical status.

It is very common in clinical practice to combine loop diuretics with thiazide diuretics, carbonic anhydrase inhibitors (e.g., acetazolamide), metolazone (thiazide like diuretic), or potassium-sparing diuretics in order to achieve sequential nephron blockade and thus overcome diuretic resistance. The addition of acetazolamide to loop diuretic therapy in patients with ADHF resulted in a higher incidence of successful decongestion but no benefit in terms of survival or rehospitalization for heart failure in a recent randomized, placebo-controlled trial (ADVOR trial) [70]. In the setting of inhibition of the Na-K-2Cl symporter by loop diuretics, sodium reabsorption is enhanced at the distal convoluted tubule [71]. This phenomenon is dealt with the combined use of thiazide diuretics resulting in greater diuresis and weight loss compared to loop diuretics alone. However, according to head-to-head comparisons between diuretics, superiority has not been proven for any of them yet [72,73]. The addition of mineralocorticoid receptor antagonists (MRA) in the context of AHF and CRS have resulted in improved natriuresis [74]. However, the ATHENA-AHF study did not show a significant benefit with the addition of MRA in the setting of AHF with diuretic resistance [75].

Diuretic resistance frequently arises during the treatment of CRS. Various responsible mechanisms have been recognized, including a reduced oral bioavailability due to intestinal edema, an accumulation of uremic toxins diminishing the secretion of diuretics into the proximal tubule, a decreased glomerular filtration of diuretics with acute reductions of GFR, and a compensatory enhancement of RAAS and SNS [69]. To overcome diuretic resistance, a combination treatment with thiazide diuretics (such as metolazone) is commonly employed. There are some data supporting the uptitration of loop diuretics as a preferred strategy over routine early addition of thiazide diuretics due to the increased prevalence of electrolyte disturbances, WRF, and mortality [76].

The use of vasodilating agents is an effective way to reduce central venous pressure (CVP) and increase net filtration pressure in the kidney. Nitroglycerin and nitroprusside are both vasodilating agents able to decrease CVP. However, neither nitrates, nitroprusside, nor nesiritide have shown robust evidence for improving clinical outcomes in AHF, except perhaps for early symptom improvement [77]. Nesiritide, a B-type natriuretic peptide, was not associated with any benefit in the rate of death and rehospitalization and had a small, nonsignificant effect on dyspnea when used in combination with other therapies. It was not associated with a WRF, but it was associated with an increase rate of hypotension. Based on these results, nesiritide cannot be recommended for routine use in patients with AHF [78].

Inotropic support is critical for maintaining perfusion pressure to vital organs but has also been associated with increased mortality in several trials. Among inotropic agents, milrinone, low-dose dopamine, and dobutamine have not shown any improvement in renal function and mortality rates. One of the most promising inotropic agents, levosimendan, has shown contradictory results. On one hand, it was associated with hemodynamic improvement and a lower mortality at 1 and 6 months but on the other hand, according to the SURVIVE and the REVIVE I and II trials, levosimendan did not improve mortality and displayed an increased risk of hypotension, cardiac arrhythmias, and death at 90 days [79,80].

Neurohormonal axis inhibition with the use of angiotensin-converting enzyme inhibitors (ACE-I) and angiotensin receptor blockers (ARB) are one of the cornerstones in the therapy of AHF. These agents improve mortality even in cases of severe renal insufficiency and/or severe CHF. Although they can acutely cause WRF, in most cases, renal function returns to baseline with the additional benefit of the long-term stabilization of kidney function [81]. Therefore, withholding neurohormonal blocking agents, in the view of a mild or temporal rise in creatinine, is not recommended [72].

Ultrafiltration (UF) is a potential useful method in parallel with diuretic therapy especially in those patients presenting with diuretic resistance and severe renal insufficiency. The CARRESS-HF study evaluated the utility of UF in ADHF with worsening renal function. The trial was terminated prematurely due to a lack of efficacy of UF and a higher prevalence of adverse events [82]. However, doubts exist concerning the use of fixed-rate UF prescribed for all patients included in the trial, because high rates of UF could be responsible for hypotension or other adverse events. The role of adjustable UF rate in ADHF was examined in a small study of 56 patients, which showed that UF treatment was associated with prolonged clinical stabilization [83]. However, larger clinical trials to support the use of UF in the area of ADHF and CRS are clearly needed. The time point at which UF should be initiated, the optimal rate of fluid removal, and the time point at which UF should be discontinued and replaced by standard therapies are some of the major concerns to be resolved [84]. In addition, different renal replacement therapy (RRT) modalities, continuous RRT (CRRT) vs. intermittent RRT, have not been compared head-to head in patients with CRS. Little is also known about the efficacy and safety of peritoneal dialysis (PD) for the treatment of acute CRS. PD has several advantages over extracorporeal dialysis as it requires less infrastructure and specialized staff, causes less hemodynamic disturbances, is associated with a lower risk of bleeding, and has showed more benefit for fluid control and the preservation of renal function compared to UF therapy [85,86]. According to a study from Thailand which included 147 patients with CRS1 during acute coronary syndrome (ACS), those who received PD as a primary treatment achieved better hemodynamic stability and survival [87]. Moreover, the hemodynamic impact of PD on venous congestion, right ventricular function, pulmonary artery systolic pressure (PASP), and clinical status was studied in 21 elderly patients with CRS and CHF. It was found that PD conferred a substantial benefit in NΥHA clinical status which was in line with an improvement in venous congestion and right ventricular systolic pressure [88]. There are presently no formal guidelines for initiating RRT in CRS patients. We believe that RRT should be initiated based on the treating physician’s clinical judgment and the current KDIGO guidelines, which state: dialysis is usually considered when there are (a) symptoms or indicators of renal failure; (b) difficulty to control volume status or blood pressure; and (c) a progressive worsening in nutritional status that is resistant to therapies. All of these indications may also apply to CRS, which means that RRT can begin well before eGFR falls below the traditional threshold of 10 mL/min/1.73 m^2^, when uremic symptoms are present or volume management and nutritional status are difficult to maintain [89].

The scientific world has largely recognized SGLT2i as an effective treatment not only for type 2 diabetes mellitus, but also for HF and CKD patients. Several trials have demonstrated its combined cardio- and renal-protective role in patients with and without type 2 diabetes mellitus. SGLT2i has been postulated to have several pleiotropic effects, including the restoration of autophagy, which may be important in the reversal of HF pathogenesis. Additional processes include the modulation of inflammatory, oxidative, and fibrotic pathways, as well as improved endothelial function and decreased epicardial adipose tissue [90,91].

Inhibiting SGLT2 in the proximal tubule lowers plasma glucose and hemoglobin A1C (HbA1C), while improving insulin sensitivity and beta-cell activity. A net calorie loss results in a loss of body weight. Aside from the effects on glucose, the combination of osmotic diuresis and natriuresis results in a decreased plasma volume (preferentially from the interstitial space) and, as a result, a decreased blood pressure. The effect of SGLT2i on tubuloglomerular feedback has also been shown to restore excess glomerular pressure and filtration [92,93,94,95]. The fast decrease in extracellular volume caused by SGLT2i is assumed to be a key mechanism behind the improved HF outcomes observed in clinical trials [92]. The use of SGLT2 inhibitors was associated with an improvement in ventricular function indices (LVEF, GLS, LVESV, LVMi, LAVi, and E/e’) in a systematic review and meta-analysis, indicating the role of SGLT2 inhibition in the reversal of cardiac remodeling [96].

They also improve renal function by both preventing and delaying the course of chronic kidney disease. SGLT2i usage has been linked to a lower glomerular pressure, a lower activity of the intrarenal renin angiotensin aldosterone system (RAAS) and sympathetic nervous system (SNS), lower inflammatory and fibrotic indicators, and an increase in hematocrit, hence reducing renal hypoxia. These effects manifest clinically as a stabilization of the estimated glomerular filtration rate (eGFR), a decreased blood pressure, and decreased albuminuria and ischemic injury [92,93].

A large body of evidence from randomized clinical studies has established SGLT2i’s effectiveness in diabetic and nondiabetic renal disease, independently of glycemic status and baseline renal function. The rise of this therapeutic class, together with the recently developed new-generation mineralocorticoid receptor antagonist finerenone, has the potential to reduce the excess burden of CKD [91].

***Key point 6:*** In the clinical setting of CRS, a multimodal therapeutic approach involves diuretic usage, neurohormonal activation inhibition, inotropic support, and extracorporeal volume management. Due to their pleiotropic effects, SGLT2i are ideal candidates to prevent or even ameliorate combined cardiac and renal dysfunction.

### 1.7. Cardiorenal Anemia Syndrome (CRAS): A New Area of Research and a Potent Therapeutic Target in CRS

CRAS is considered to be a pathological triangle in which the dysfunctional interplay between the kidney and the heart may lead to the development of anemia. When anemia appears, a vicious cycle commences, where HF and renal dysfunction cause anemia, which in turn will worsen the first two conditions and will unfavorably affect morbidity and mortality [97]. Anemia is frequently observed in patients with CHF and is associated with an increased risk of mortality [98]. The prevalence of anemia, varying from 14% to 70%, increases in parallel with the severity of CHF, the CKD stage, and patients’ age, while the treatment of anemia leads to an improvement in cardiac and renal function as well as to less hospitalizations for HF [99].

According to the optimize-HF registry, anemia is associated with a 30% increase in all-cause mortality and morbidity [100]. The combined impact of CRS and anemia on mortality was evaluated by the ANCHOR trial. In that trial, it was shown that very high (≥17 g/dL) or very low (<13 g/dL) hemoglobin levels independently predicted an increased risk of death and hospitalization in CRS with both preserved and reduced systolic function [101]. The increased mortality in anemic CHF patients is partially explained by the hemodynamic and nonhemodynamic responses caused by the decreased oxygen supply to the tissues. These anemia responses include an increased left ventricle workload and output, an increased activity of the renin–angiotensin–aldosterone system and sympathetic nervous system, a retention of salt and water, a reduced GFR and renal blood flow, which, jointly, result in a deterioration of the HF status and adverse outcomes [102].

The development of CRAS is multifactorial and several contributing risk factors have been recognized. Advanced age, a low body mass index, diabetes mellitus, a reduced left ventricular ejection fraction (LVEF), the omission of renin–angiotensin system inhibitors, and the use of intravenous loop diuretics are independently associated with CRAS [97,103]. Furthermore, deficiencies in vitamin B12 and folic acid, iron deficiency, blood losses caused by aspirin and anticoagulants, an expansion of plasma volume and hemodilution, increased inflammation causing reduced erythropoietin (EPO) production and action, concomitant renal insufficiency, poor nutritional status, intestinal malabsorption due to significant edema are some of the conditions leading to anemia in HF patients.

Anemia of CKD has several underlying mechanisms. Among the most important ones are an inadequate synthesis of EPO, a limited availability of iron for erythropoiesis, increased hepcidin, reduced EPO receptors, and finally, the use of ACE inhibitors and ARBs [102,104,105]. In a large cohort study of patients with HF, it was shown that EPO levels were usually increased due to chronic inflammation resulting in a resistance of the bone marrow for EPO and the inhibition of erythropoiesis [104,106]. Concurrently, iron metabolism was also altered due to the increased production of hepcidin, a hormonal response triggered by the chronic inflammatory status, reducing iron absorption from the gastrointestinal tract and its bioavailability for hemoglobin synthesis [107].

Currently, there are no evidence-based recommendations for managing patients with CRAS. The treatment of these patients is multifaceted as a concomitant control of anemia, renal dysfunction, and heart failure is needed. Kidney Disease Improving Global Outcomes (KDIGO) organized an international conference and concluded that erythropoiesis-stimulating agents (ESAs) had no impact on the prevention or treatment of HF in patients with CKD [108,109]. On the contrary, the treatment of patients with chronic HF and iron deficiency, with or without anemia, with intravenous iron, resulted in improved functional capacity, eGFR, and symptoms according to the findings of several studies [110,111].

Intravenous iron and ESAs are the cornerstone therapy for anemia treatment in patients with CKD [112]. However, for patients with anemia and HF, the use of ESAs is currently not recommended due to their adverse outcomes, leaving only intravenous iron as the recommended therapy for these patients. Intravenous iron therapy has been shown to improve iron parameters in parallel with improvements in NYHA functional status, 6 min walk test (6MWT), and quality of life (QOL) in patients with HF and iron deficiency, with or without anemia or CKD [110,113,114,115].

ESA therapy has been associated with reductions in LV mass and wall thickness and an improvement in renal parameters [116]. However, treatment with darbepoetin alfa did not improve clinical outcomes in patients with mild to moderate anemia and systolic heart failure and provoked thromboembolic events [116]. The American College of Cardiology Foundation, the Heart Failure Society of America, and the European Society of Cardiology recommended against the use of ESAs for the treatment of anemia in HF patients [117]. Trials of ESAs in patients with anemia and CKD have demonstrated an increased risk of cardiovascular events associated with higher Hb targets [118,119,120]. Overall, ESA therapy is prescribed in a small proportion of patients with CRAS based on the KDIGO recommendations for the treatment of anemia in CKD patients [118,121]. Therefore, intravenous repletion of iron stores can be a sufficient single therapy for anemia in CRAS patients with an additional benefit on HF (see Table 1).

Hypoxia-inducible factor prolyl hydroxylase inhibitors (HIF-PHIs) represent a novel class of drugs for the management of anemia in CKD and CRAS. These inhibitors promote physiological EPO production by inhibiting the prolyl hydroxylase enzymes that are responsible for the degradation of the hypoxia-inducible factors (HIF), the transcription factors that induce expression of EPO in the kidneys and the liver. Apart from their impact on EPO, HIFs drive a coordinated response resulting in increased iron uptake and decreased hepcidin levels, leading to a more effective mobilization and usage of iron. Clinical trials with HIF-PHIs have shown a decrease in hepcidin and ferritin levels and increase in total iron binding capacity and usage of iron for erythropoiesis [122]. There have been some recent trials with different oral inhibitors (vadadustat, daprodustat, desidustat) that showed maintenance or improvement of anemic status in patients with CKD and anemia [123,124]. However, HIFs have additional effects that are not necessarily beneficial due to their ability to affect multiple organs and cellular functions and having an impact on angiogenesis, tumor growth, and glucose metabolism [123,124]. Therefore, the long-term safety of these agents must be further investigated in future studies.

***Key point 7:*** Given the limited therapeutic approaches available, CRAS poses a clinical difficulty. A fraction of CRS patients with severe CKD may benefit from tight anemia management with ESAs. Iron store replenishment may be beneficial in the majority of CRAS patients.

## 2. Conclusions

A CRS classification system that assesses the underlying organ damage and its temporal sequence will assist clinicians in providing appropriate therapy to the proper patient. Furthermore, a combination of several biomarkers (both cardiac and renal) must be established in order to identify patients who are at higher risk of developing a more severe form of CRS. Serum chloride and CNP appear to be potential indicators, but additional research is needed before they can be used in clinical practice. CRS management is still complicated due to the intricacies of decongestive therapy and inotrope selection, the management of diuretic resistance, and the best timing and dose of ultrafiltration therapy. Anemia in the context of cardiac and renal failure comprises a complex triad that should always be addressed.

## Figures and Tables

**Table 1:** Important Key Points in CRS Diagnosis, Classification, and Management.

Novel methods and biomarkers are required for accurate clinical classification of CRS.
Artificial intelligence support systems and clinical algorithms may be used to identify patients with CRS who are at risk of adverse outcomes.
Panel of novel plasma and urine biomarkers for risk stratification and for the distinction of WRF from true AKI.
Incorporation of improved methods of assessing venous congestion (VExUS) into routine clinical practice
Volume and Neurohormonal Control, SGLT2i, Inotropic support, Ultrafiltration, Iron repletion, Finerenone

## Data Availability

Not applicable.
